# Acceptability of a Plasticity-Focused Serious Game Intervention for Posttraumatic Stress Disorder: User Requirements Analysis

**DOI:** 10.2196/11909

**Published:** 2019-04-16

**Authors:** Matthew Jones, Alena Denisova, Stephen Mitchell, Tom Owen

**Affiliations:** 1 Health Services Research Medical School Swansea University Swansea United Kingdom; 2 Department of Computer Science College of Science Swansea University Swansea United Kingdom

**Keywords:** PTSD, mobile applications, neuronal plasticity, cognitive behavioral therapy

## Abstract

**Background:**

Trauma-focused cognitive behavioral therapy (TF-CBT) is a first-line treatment for posttraumatic stress disorder (PTSD). Despite a solid evidence base, TF-CBT response and attrition rates vary considerably. Plasticity-focused interventions, including the use of serious games, have the potential to improve TF-CBT response and treatment retention.

**Objective:**

The aim of this study was to assess the acceptability of a mobile phone–delivered plasticity-focused serious game to improve response to TF-CBT for PTSD, and carry out a user requirements analysis should the development of a prototype be warranted.

**Methods:**

We conducted 2 one-to-one interviews (n=2), one focus group involving service users who had received a diagnosis of PTSD (n=3) and one focus group involving psychological trauma service clinicians (n=4).

**Results:**

We found that the concept of a plasticity-focused mobile phone intervention for PTSD is acceptable to patients and clinicians. Service users and clinicians both believed that the usage should be guided by a therapist, and both contributed useful inputs regarding the audiovisual aspects of the proposed serious game. It was accepted that the game would not be suitable for all patients and that clinicians would need to appropriately prescribe the usage of the game.

**Conclusions:**

The findings highlight the acceptability of the proposed serious game and clarify the user requirements for such an intervention. It is the intention of the authors to carry out a user experience evaluation using a prototype serious game in a clinical population.

## Introduction

### Background

In the absence of UK estimates, lifetime prevalence of posttraumatic stress disorder (PTSD) in the United States has been estimated to be between 6.8% and 7.8% [1]. This disorder is characterized by intrusive memories of traumatic events, nightmares, and avoidance of event-related stimuli [2]. In the United Kingdom, trauma-focused cognitive-behavioral therapy (TF-CBT) is the first-line treatment for PTSD [3]. TF-CBT refers to a range of evidence-based psychotherapeutic techniques for the treatment of psychological trauma. Despite a strong evidence base and wide implementation of TF-CBT, non-response and attrition rates vary widely, in some circumstances exceeding 50% [4,5]. Contributory factors that reduce optimal response to therapy in PTSD are varied, such as many possible social and behavioral factors [4]. One possible contributory variable is neural deficit associated with PTSD. Functional imaging studies suggest that reduced frontomedial neural network connectivity (eg, reduced prefrontal modulation of the amygdala) and volumetric abnormality of medial limbic structures are associated with PTSD psychopathology [6-8].

### Neural Correlates of Posttraumatic Stress Disorder

According to the theoretical frameworks underpinning TF-CBT, frontomedial neural connectivity is thought to play a critical role in regulating computational processes that are integral to processing trauma memories. These processes include pattern identification, separation, and completion [9], which allow for complete retrieval and successful discrimination between contextually similar episodic or event memory formations based on familiar stimuli. Difficulty in completing these processes is a characteristic of PTSD. For example, someone who experiences posttraumatic symptoms in relation to the trauma of surviving a house fire, may experience involuntary recall of traumatic memory in response to the sight and smell of a bonfire.

There is also evidence of intrinsic medial temporal lobe (MTL) connectivity contributing to the successful processing of fear-related memory [10] and the formation of egocentric episodic memory representations [11]. Reduced neural connectivity in the MTL, especially in relation to the strong efferent connections between the retrosplenial cortex and the hippocampus, may have negative clinical implications for PTSD patients engaging in TF-CBT. This is because a core component of TF-CBT, known as *reliving*, is a form of in vitro exposure that involves the guided retrieval of episodic memory of traumatic incidents from an egocentric viewpoint in vivid detail.

### Objective

Several researchers have found functional and structural plastic changes in neural circuits following the prolonged use of commercial video games and have queried the potential for therapeutic application [12-14]. Neuroimaging and behavioral data suggest that frontomedial circuitry is implicated in generating short-term memory representations related to way-finding and memory-based decision-making tasks [15,16], similar to the use of salient landmarks to aid navigation of novel three-dimensional (3D) environments [17]. In addition, mechanics such as switching between a first- or third-person perspective and an aerial map perspective of a virtual environment have been associated with recruitment of frontomedial circuitry [12]. On the basis of these data, it appears possible that a specifically designed video game (serious game) could encourage plastic changes in frontomedial neural circuitry by presenting the player with tasks known to recruit this neural network. For example, a video game could present a player with a two-dimensional aerial map representation of a novel 3D environment, inside which several objects were placed. The player could then be asked to memorize the location of each object, choose an order to collect the objects, then navigate the environment, and collect each object in the order they have chosen, aided by salient environmental landmarks.

This study aimed to find out the following:

- If a plasticity-focused serious game intervention designed to aid response to TF-CBT in PTSD patients would prove an acceptable concept to service users and providers?
- What would be the user requirements for such an intervention to be feasible and accessible?

## Methods

The authors conducted a focus group (n=3) and 2 semistructured interviews (n=2) with service users and, in addition, conducted a focus group with psychological trauma service clinicians (n=4).

### Participants

The recruited number of eligible participants was 9. In total, 5 participants were service users and 4 were trauma service clinicians. Among them, 1 service user participant was a female, and the remaining 4 were males. Mean age for service user participants was 59.8 years with the age range between 53 and 68 years. The duration of PTSD symptoms reported by service user participants ranged from 3 to 30 years. A total of 3 participants had experienced combat-related trauma. The nature of trauma experienced by the other service users was unknown. The clinical roles represented by the trauma service clinicians (n=4) were 1 trainee clinical psychologist, 1 assistant psychologist, 1 specialist clinical psychologist, and 1 consultant clinical psychologist. Principal inclusion and exclusion criteria are presented in Textbox 1.

**Principal inclusion and exclusion criteria.**

- Service users
  - Inclusion criteria: aged 18 years and above; previous or current diagnosis of posttraumatic stress disorder (PTSD); in frequent and regular contact with service clinicians for treatment or other services, eg, mentoring
  - Exclusion criteria: aged under 18 years; inability to provide informed consent to participate
- Clinicians
  - Inclusion criteria: aged 18 years and above; currently working clinically with patients with PTSD
  - Exclusion criteria: not working directly with patients with PTSD; unqualified or trainee clinicians without ongoing clinical supervision

**Topic guide: participants and domains of inquiry.**

- Service users
  - Current mobile phone capabilities
  - Coping strategies employed by service users (and those which involve mobile phone apps or Web-based resources)
  - Acceptability of the proposed intervention (including concerns)
  - Views on game presentation (in terms of graphics and audio)
- Clinicians
  - Acceptability of the proposed intervention (including concerns)
  - Views on game presentation (in terms of graphics and audio)

### Materials

A topic guide was developed by the authors and used for both the focus groups and interviews. Open-ended questions with prompts addressed a total of 5 broad domains of inquiry (as summarized in Textbox 2). All topics were explored in each interview and focus group. Interviews lasted between 30 and 60 min, and focus groups lasted between 90 and 120 min. Demographic data, including age and gender, were requested from service user participants only. We sought permission from all participants to make live written notes and audio-record interviews and focus groups on digital voice recorders placed on a table, around which participants and researchers sat. All audio and written data were transcribed verbatim on a later date.

### Procedure

To identify and engage members of the target population for recruitment into the study, the authors approached several organizations, including third sector organizations and National Health Services. A branch of a national charity offering mental health mentoring and practical support to military veterans in South Wales, responded promptly and positively to our queries. One-to-one interviews were carried out at the charity’s premises during April 2017. We decided not to include the name and location of this service in the interest of the confidentiality and anonymity of study participants. The psychological trauma service at Springfield University Hospital in Tooting, London, also responded positively and promptly to our enquiries. Focus groups involving patients and clinical staff were carried out here in June 2017.

### Data Analysis

Data were thematically analyzed, summarized, and described in relation to our domains of inquiry. As the interviews were relatively unstructured and we sought to assess acceptability rather than develop a theory of theoretical framework, we attempted to calculate interrater reliability.

## Results

### Current Service User Mobile Phone Capabilities

Of the 5 service user participants, 4 owned a mobile phone and 1 owned an older generation mobile phone. Of the 4 participants who owned a mobile phone, 3 owned a mobile phone running Google’s Android operating system and the other used a phone running Apple’s iOS. Participants’ mobile phones were capable of running software apps, and all reported social media apps that were their favored and most frequently used apps. These included Facebook, Facebook Messenger, and WhatsApp.

### Existing Coping Strategies Reported by Service Users

All participating service users reported reliance on particular lifestyle-related strategies to cope with PTSD symptoms. The majority of participants reported social support (through either friends or family or organized support groups) and engagement in outdoor activities, including cycling and dog walking. All but one participant reported using mobile apps in seeking support from others (eg, Facebook, WhatsApp, short message service text messaging, phone).

All participating service users reported that PTSD had adversely affected their memory. Participants gave examples of impaired episodic and prospective memory capacity, such as frequent examples of the *doorway effect* and forgetting seemingly meaningful upcoming or past events, particularly when stressed. Out of the 5 service users, 3 participants did not use any memory-specific coping strategies, whereas 1 participant reported using mobile phone to create calendar reminders, and 1 reported writing things down using a pen and paper. Walking and cycling were reported to be helpful activities by 2 participants, and 1 reported using Google Maps for planning cycling routes. All but 1 had previous TF-CBT; all reported current medication with selective serotonin reuptake inhibitors.

No participants had used Web-based resources to help them cope with PTSD symptoms. Only 1 of 5 participants reported having used an app called PTSD Coach [18] released by the United States Department of Veterans Affairs. The app focuses on education around PTSD and symptom tracking and is marketed as a therapeutic tool. They described the app as unhelpful and were no longer using it. No participant reported actively using general health or well-being–related apps. Usage of relaxation and meditation apps were reported by 2 participants (these were the freely available *Stop, Breathe and Think* and *Smiling Mind* Android apps) [19,20], which included scripted meditation and relaxation exercises. Both participants described these as unhelpful, with 1 participant describing the sensation of being *out of control* when using the apps, as they were anxiety inducing.

### Service Users’ Reported Acceptability of the Proposed Serious Game

All participants described themselves as being willing to try the proposed serious game intervention. Only 1 participant was less enthusiastic about using the app along with therapy than before therapy, and 1 was concerned that using the app would interfere with therapy rather than enhance it; however, these concerns could be alleviated if assurance of the game’s efficacy was provided by a clinician. All participants stated that they would be more confident about using the app under the guidance of a clinician.

Of the 5 participants, 3 stated that they would be unlikely to use the app at times when they were experiencing acute symptoms of PTSD, for example, experiencing a flashback or symptoms of panic. Only 1 participant described long periods of inactivity alluding to depressive symptoms as potential barrier to using the app. All agreed that the app would have to be easy to use and intuitive in its design. All participants expressed concern that if the game were too difficult, they may become frustrated and cease using it relatively quickly. Should this not be the case, 2 participants stated that they would be willing to use the app for 30 min every day over a prolonged period (defined by the interviewer as between 3 and 6 weeks). The remaining 3 participants stated they would be willing to use the app for 30 min every other day over the same period. All participants were enthusiastic about the prospect of patients using the serious game while on a waiting list. They described positive implications for self-efficacy:

...you’re not just waiting for someone to help you then, you are doing something [to help] yourself.

All participants were positively inquisitive about the concept behind the proposed serious game, especially the fact that the game would not include a psychotherapeutic component to address PTSD or PTSD-related symptoms directly. No participant reported any concerns or negative attitudes toward the concept underpinning the proposed serious game when prompted, for example:

What do you think of the serious game concept?

Do you think this could work for you?

### Service Users’ Views on the Proposed Serious Game's Graphics and Audio Components

In terms of graphical and audio design features that would be beneficial to the user experience, all participants favored an expansive outdoor environment. Water, in the form of rivers and lakes, including the sound of naturally running water was described as desirable by 4 participants. However, beaches were described as reminiscent of combat trauma and therefore especially undesirable by 2 of the 4 participants. Desert landscapes were also described as undesirable. Woods and greenery were described as desirable by 4 participants, and 1 participant described the ideal aesthetic to be that of a garden. The need for the player to be able to choose different seasons for the environment before the play was expressed by 3 participants. No participant desired the inclusion of human characters in the environment; however, 3 enthusiastically favored the inclusion of wildlife such as dogs, birds, and horses.

At the time of the focus groups and interviews, the researcher would describe the need for novel landmarks to be located in the environment and ask the participants how they would like these to be represented. In response to this query, mountains and trees were described as ideal landmarks by all participants. Man-made structures were described by 3 participants, with Buddhist temple or similar architecture being the favored choice.

### Clinicians’ Reported Acceptability of the Proposed Serious Game

As with service user participants, clinicians were positively inquisitive about the concept behind the proposed serious game. Clinicians were more likely to inquire about the theoretical basis for the intervention. No clinician reported any concerns about potential adverse effects; however, all participants favored clinician-guided use of the game as opposed to independent use. It was the prevailing view that a significant proportion of patients would require coaching in using the game with adequate frequency.

### Clinicians’ Views on the Proposed Serious Game's Graphics and Audio Components

When discussing the sound and graphics content of the game, all participants agreed that the game must avoid sound and graphic content likely to trigger symptoms of PTSD. All agreed that the more realistic the environment was, the more difficult this task became, and so a *cartoon-like* or abstract appearance for the environment was favored. It was accepted that creating an environment that guaranteed not to trigger symptoms of PTSD in any player would be infeasible. However, it was accepted that creating an environment that was unlikely to trigger PTSD symptoms for the majority of users was feasible, especially if the environment was customizable and the game was carefully prescribed.

Clinicians stated that the game should match the patient’s abilities:

...if the game is too boring, the patient would disengage, weakening the effect of the experimental manipulation.

Service users, on the other hand, were more concerned with the amount of concentration required to sustain while playing the game; if they had to concentrate too hard, they could get frustrated and give up. This balance of skill and difficulty is referred to as a component of *flow* theory and is frequently referenced when designing games for educational and clinical purposes [21].

Clinicians raised the issue of increasing the numbers of asylum seekers seeking help for PTSD in the United Kingdom in recent years. As many of these service users may have difficulty understanding English, the game interface must be intuitive and include very little text guidance. Translating the game into many different languages would present a less feasible option, given the available funding and resources. In addition, in the clinician’s experiences, many patients who had arrived in the country seeking asylum had come from Middle Eastern and North African countries and had experienced trauma not only in the countries from which they were escaping but also during their perilous journeys to the United Kingdom. For this reason, graphics of beaches, large expanses of open water, boats, lorries, and desert landscapes were deemed undesirable.

## Discussion

### Principal Findings

We captured qualitative data regarding the acceptability of a plasticity-focused serious game to aid response to TF-CBT for PTSD from a small sample of service users and clinicians. These data suggest that the concept of such an intervention is acceptable to both groups and can be made accessible to the target population who mostly have access to capable hardware. Care must be taken in terms of graphical and audio content to ensure that the game does not elicit trauma symptoms in service users and is accessible and easy to use. This represents a significant design challenge given the highly varied clinical population in question. It was the view of the service users and clinicians that the game usage should be guided by a therapist. The issue of negative affect influencing motivation and poor prospective memory in PTSD patients reinforced the need for therapist involvement. It was also accepted that the game would not be suitable for all patients, and so the clinicians would have to use their knowledge and expertise to appropriately prescribe usage of the game.

### Limitations

This study was limited by a small and opportunistic sample, most notably missing asylum-seeking representatives. Participants had received a diagnosis of PTSD by specialist trauma clinicians. Details of individual’s diagnosis, including the nature of the trauma or traumas, or the symptom severity at the time of participation, were not available to the researchers. In addition, we did not collect data related to spiritual or religious beliefs, which could have helped put in to context the preference for Buddhist style structures in the virtual environment.

### Future Steps

We propose to complete user-experience testing with service users and clinicians using a prototype video game running on a medium-specification Android device. We will measure readiness to use a digital intervention such as Hippocampal and Prefrontal Plasticity Inducement Application (HAPPIA), using a previously validated method of assessment such as the eHealth Readiness Scale [22], and include a user satisfaction questionnaire to supplement our qualitative data collection. We will also formally measure aspects of participant’s individual pathology using validated tools. For example, we will measure engagement in existing coping strategies for trauma symptoms using the Trauma Coping Self-Efficacy scale [23]; the Life Events Checklist will be used to assess the nature of experienced trauma events [24]; and the Posttraumatic Stress Disorder Checklist for the Diagnostic and Statistical Manual of Mental Disorders, fifth edition will be used to measure symptom severity [25]. Data from this testing will be used to refine the design of the software and further inform the choice of hardware used to deliver the intervention before clinical evaluation. Screenshots from the in-development prototype informed by the qualitative data reported here are presented below. The prototype dubbed “HAPPIA” has begun development using the Unity software engine developed by Unity Technologies and is optimized to run on medium-spec Android devices including mobile phones and tablet personal computers.

Following further refinement and development of the HAPPIA app by way of user-experience testing, we will seek to evaluate HAPPIA as a clinical intervention by carrying out a randomized controlled trial. Patients awaiting a course of TF-CBT will be randomly assigned to HAPPIA plus treatment as usual (TAU) or TAU alone. Symptom severity at the outset of treatment, at cessation of treatment, and at follow-up will be measured using validated psychometric measures and compared among groups. Attrition rates and changes in psychotropic medication (eg, anxiolytics) dosage will also be compared among groups. We will capture demographic data, including age and gender, for clinical participants. HAPPIA will record game-related data, including time spent playing the game, time taken to complete tasks, and efficiency of task completion for within-group analysis.

## Abbreviations

3D: three-dimensional
HAPPIA: Hippocampal and Prefrontal Plasticity Inducement Application
MTL: medial temporal lobe
PTSD: posttraumatic stress disorder
TF-CBT: trauma-focused cognitive-behavioral therapy
